# Integrative study of EZH2 mutational status, copy number, protein expression and H3K27 trimethylation in AML/MDS patients

**DOI:** 10.1186/s13148-021-01052-2

**Published:** 2021-04-12

**Authors:** Julia Stomper, Ruth Meier, Tobias Ma, Dietmar Pfeifer, Gabriele Ihorst, Nadja Blagitko-Dorfs, Gabriele Greve, Dennis Zimmer, Uwe Platzbecker, Anne Hagemeijer, Annette Schmitt-Gräff, Michael Lübbert

**Affiliations:** 1 Department of Medicine I (Hematology, Oncology and Stem Cell Transplantation), Medical Center – University of Freiburg, Freiburg, Germany; 2 Clinical Trials Unit, Faculty of Medicine, Medical Center - University of Freiburg, Freiburg, Germany; 3 Institute for Immunodeficiency, Center for Chronic Immunodeficiency (CCI), Medical Center - University of Freiburg, Freiburg, Germany; 4 German Cancer Consortium (DKTK) and German Cancer Research Center (DKFZ), Partner Site Dresden, Dresden, Germany; 5 Medical Department-Hematology and Cell Therapy, Medical Oncology, Hemostaseology, University of Leipzig Medical Center, Leipzig, Germany; 6 University of Leuven, Leuven, Belgium; 7 University of Freiburg, Freiburg, Germany; 8 German Cancer Consortium (DKTK) and German Cancer Research Center (DKFZ), Partner Site Freiburg, Freiburg, Germany; 9 Faculty of Medicine, University of Freiburg, Freiburg, Germany

**Keywords:** Acute myeloid leukemia, EZH2, Mutations, Protein expression, H3K27 trimethylation, Promoter methylation, Survival

## Abstract

**Background:**

Mutations in the *EZH2* gene are recurrently found in patients with myeloid neoplasms and are associated with a poor prognosis. We aimed to characterize genetic and epigenetic alterations of *EZH2* in 58 patients (51 with acute myeloid leukemia and 7 with myelodysplastic or myeloproliferative neoplasms) by integrating data on *EZH2* mutational status, co-occurring mutations, and *EZH2* copy number status with EZH2 protein expression, histone H3K27 trimethylation, and *EZH2* promoter methylation.

**Results:**

*EZH2* was mutated in 6/51 acute myeloid leukemia patients (12%) and 7/7 patients with other myeloid neoplasms. *EZH2* mutations were not overrepresented in patients with chromosome 7q deletions or losses. In acute myeloid leukemia patients, *EZH2* mutations frequently co-occurred with *CEBPA* (67%), *ASXL1* (50%), *TET2* and *RAD21* mutations (33% each). In *EZH2*-mutated patients with myelodysplastic or myeloproliferative neoplasms, the most common co-mutations were in *ASXL1* (100%), *NRAS*, *RUNX1*, and *STAG2* (29% each). *EZH2* mutations were associated with a significant decrease in EZH2 expression (*p* = 0.0002), which was similar in patients with chromosome 7 aberrations and patients with intact chromosome 7. An association between EZH2 protein expression and H3K27 trimethylation was observed in *EZH2*-unmutated patients (*R*^2^ = 0.2, *p* = 0.01). The monoallelic state of *EZH2* was not associated with *EZH2* promoter hypermethylation. In multivariable analyses, *EZH2* mutations were associated with a trend towards an increased risk of death (hazard ratio 2.51 [95% confidence interval 0.87–7.25], *p* = 0.09); similarly, low EZH2 expression was associated with elevated risk (hazard ratio 2.54 [95% confidence interval 1.07–6.04], *p* = 0.04).

**Conclusions:**

Perturbations of EZH2 activity in AML/MDS occur on different, genetic and non-genetic levels. Both low EZH2 protein expression and, by trend, *EZH2* gene mutations predicted inferior overall survival of AML patients receiving standard chemotherapy.

**Supplementary information:**

The online version contains supplementary material available at 10.1186/s13148-021-01052-2.

## Background

Enhancer of Zeste Homolog 2 (EZH2), the catalytic domain of Polycomb Repressive Complex 2 (PRC2), catalyzes the repressive mark of trimethylation of histone H3 lysine 27 (H3K27me3) [1]. The *EZH2* gene is located on chromosome 7q36.1, a region frequently deleted in acute myeloid leukemia (AML), myelodysplastic syndromes (MDS), and myeloproliferative neoplasms (MPN) [2]. MDS and AML patients with monosomy 7 or 7q (-7/del(7q)) were found to have reduced *EZH2* mRNA expression levels compared to patients without these alterations [3, 4]. The role of *EZH2* in tumorigenesis appears to be context-dependent, since both *EZH2* overexpression and loss of function are associated with different types of cancer [5]. In patients with MDS or MDS/MPN, recurrent loss-of-function *EZH2* mutations [6–8] are associated with a poor prognosis [9], and *EZH2* is considered by many to act as a *bona fide* tumor suppressor gene [10, 11]. In AML, the prevalence of *EZH2* mutations is lower and less well studied [12]. Regarding the transformation of MDS and MPN to AML, loss of EZH2 function also appears to play an ambiguous role, being able to attenuate and promote leukemic transformation depending on the disease context and cooperating mutations [11, 13, 14]. While EZH2 inhibitors are already being evaluated in phase 1 and 2 clinical trials in different solid tumors and B cell lymphoma [15], not only in MDS [16] but also in AML, EZH2 is increasingly under study as a potential therapeutic target. Yet, due to its complex function, thus far the mechanisms of action of proposed applications differ widely, ranging from preventing EZH2 degradation in order to overcome chemoresistance to inhibiting the PRC2 components EZH1 and -2 to reduce quiescent leukemia stem cells [4, 17].

In the present study, we analyzed a cohort of 58 patients (mostly AML). Since potential mechanisms of *EZH2* perturbation involve mutations, allelic loss, and promoter hypermethylation, we sought to determine those parameters, their relationship, and prognostic impact, confirming the overall worse outcome in the presence of *EZH2* mutations and decreased EZH2 expression.

## Results

### EZH2 mutational status, co-occurring mutations, and chromosome 7 loss in AML/MDS patients

**Fig. 1:**
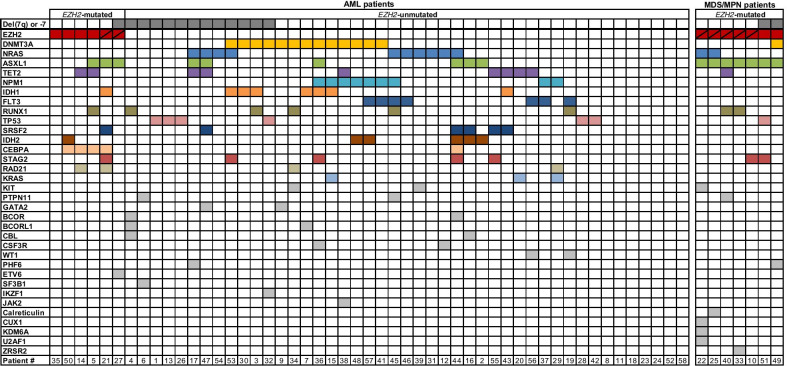
Mutations and chromosome 7 status in 58 patients with myeloid neoplasms. Each column represents one patient. Patients are grouped by disease (AML or MDS/MPN), the presence or absence of *EZH2* mutations (slash indicates two mutations), and the presence or absence of chromosome 7 aberrations

In the cohort of 58 selected patients with myeloid neoplasms (see Table 1, 2, and Additional File 1: Table S1 for patient characteristics), the *EZH2* gene showed mutations (mostly missense or nonsense) in 6/51 AML patients (12%) and 7/7 non-AML patients (selected specifically for this genotype, hence 100%, Table 2), and was unmutated in 45/51 AML patients (88%). The *EZH2* mutation rate in the different AML subgroups was 12% (3/25) in de novo AML, 9% (2/22) in AML with myelodysplasia-related changes (MRC), and 25% (1/4) in therapy-related AML (t-AML). Two of 6 *EZH2*-mutated (mut) AML patients and 5/7 MDS/MPN patients had 2 mutations within the *EZH2* gene (Fig. 1, Table 3). The median variant allele frequency (VAF) of *EZH2* mutations was 21.5% (range, 9–63%) in AML and 45% (range 7–54%) in MDS/MPN patients.

**Table 1:** Patient characteristics

	AML	Myeloid Neoplasms (Non-AML)
Total	51	7
Subtype (%)	AML with recurrent	MDS-MLD: 2
	genetic abnormalities: 20 (39)	MDS-EB-2: 1
	AML-MRC: 22 (43)	CMML-2: 1
	t-AML: 4 (8)	MDS/MPN: 2
	AML, NOS: 5 (10)	CNL: 1
Age (years; median and range)	63 (20–86)	69 (42–84)
Sex		
Male	25	4
Female	26	3
*EZH2*-mutated patients (%)	6 (12)	7 (100)
Mutations per patient (median and range)	3 (0–7)	4 (3–7)
Del(7q) or -7 (%)	13 (25)	2 (29)
WBC (× 10^9^/L; median and range)	10.4 (0.6–192.6)	18.4 (1.6–126.5)
BM blasts (%; median and range)	63 (10–100)	4 (1–16)
Treatment		
Induction chemotherapy (%)	31 (61)	0
HSCT (%)	27 (53)	4 (57)
Non-intensive therapy (%)	13 (25)	3 (43)

*AML-MRC* acute myeloid leukemia with myelodysplasia-related changes, *BM* bone marrow, *CMML* chronic myelomonocytic leukemia, *CNL* chronic neutrophilic leukemia, *HSCT* hematopoietic stem cell transplantation, *MDS-EB* myelodysplastic syndrome with excess blasts, *MDS-MLD* MDS with multilineage dysplasia, *MPN* myeloproliferative neoplasm, *NOS* not otherwise specified, *WBC* white blood cells, *t-AML* therapy-related AML

**Table 2:** Clinical characteristics of *EZH2*-mutated patients

Patient #	Sex	Age (years)	Disease subtype	Karyotype	Del(7q) or -7	% monosomy 7 cells (FISH)	WBC (× 10^9^/L)	PB blasts (%)	Treatment
*AML patients*									
14	m	29	AML with recurrent genetic abnormalities	46,XY [22]	No	0	16.4	64	Induction chemo, HSCT
35	f	84	AML-MRC (history of MDS)	48,XX,der(1)t(1;5)(p36;?), + der(1)t(1;5)(p36;?),der(5)t(5;11)(p15;q23)t(1;5)(p36;q15),der(11)t(5;11)(?;q23),del(12)(p12p13), + 19 [5]/49,idem, + 8 [9]/46,XX [6]	No	0	2.66	2	LDAC, tranylcypromine, ATRA
50	m	71	AML, NOS	46,XY [21]	No	0	101.73	26	Induction chemo, HSCT
5	f	74	AML with recurrent genetic abnormalities	46,XX [20]	No	0	31.23	49	Induction chemo, HSCT
21	m	71	t-AML	46,XY,del(12)(p12p13) [30]	No	0	4.22	54	DAC
27	m	68	AML-MRC	45,XY,inv(3)(q21.3q26.2),-7 [20]	Yes	40	2.01	5	DAC, HSCT
59	m	75	AML, NOS (history of MPN)	47,XY, + 8 [2]	No	0	3.6	5^†^	DAC
60	m	61	AML-MRC (history of MDS)	46,XY [20]	No	0	9.5	24^†^	DAC, ATRA
MDS/MPN patients									
51	m	81	MDS-MLD	45,XY,-7,der(17)t(1;17)(p36;p12) [6]/46,XY [14]	Yes	54	1.61	0	AZA
49	f	42	MDS-MLD	45,XX,-7 [9]/46,XX [13]	Yes	16	2.0	3	Upfront HSCT
25	m	82	MDS/MPN (progression to AML)	46,XY,del(17p13),i(17q11) (40%)*	No	0	39.72	10	HU, AZA
22	f	61	CNL	46,XX	No	0	71.14	0	HU, HSCT
33	m	84	CMML-2	47,XY, + 8 [19]/46,XY [1]	No	0	18.4	2	DAC
40	f	69	MDS/MPN	47,XX, + 8 [11]	No	0	126.45	6	HU, HSCT
10	m	68	MDS-EB-2	47,XY, + 8 [12]/46,XY [8]	No	0	1.67	0	AZA, HSCT

*AML* acute myeloid leukemia, *ATRA* all-*trans* retinoic acid, *AZA* azacitidine, *CMML* chronic myelomonocytic leukemia, *CNL* chronic neutrophilic leukemia, *DAC* decitabine, *FISH* fluorescence in situ hybridization, *HSCT* hematopoietic stem cell transplantation, *HU* hydroxyurea, *LDAC* low dose cytarabine, *MDS-EB* myelodysplastic syndrome with excess blasts, *MDS-MLD* MDS with multilineage dysplasia, *MPN* myeloproliferative neoplasm, *MRC* myelodysplasia-related changes, *NOS* not otherwise specified, *PB* peripheral blood, *t-AML* therapy-related AML, *WBC* white blood cells

*FISH analysis

^†^Bone marrow blasts

**Table 3:** Mutation details, EZH2 IHC score, and H3K27 trimethylation level of *EZH2*-mutated patients

Patient #	*EZH2* mutation	Mutation type	Clinical significance	VAF (%)	EZH2 IHC score	H3K27me3 level (%)
*AML patients*						
14	c.458 A > G, p.Tyr153Cys	Missense	Yes	14	3	90
35	c.2187dupT, p.Asp730*	Frameshift	Unknown	15	2	90
50	c.100C > T, p.Arg34*	Nonsense	Yes	44	1	50
5	c.857G > T, p.Cys286Phe	Missense	Yes	46	0	70
21	c.637delC, p.Arg213Alafs*28; c.1036G > T, p.Glu346Ter	Frameshift Nonsense	Unknown Unknown	11 9	1	N/A
27^†^	c.1768 T > A, p.Cys590Ser; c.2022G > T, p.Leu674Phe	Missense Missense	Yes Unknown	28 63	1	N/A
59	c.2221dupT, p.Tyr741Leufs*22	Frameshift	Unknown	78	N/A	N/A
60	c.735dupA, p.Glu259*	Nonsense	Unknown	90	N/A	N/A
*MDS/MPN patients*						
51^†^	c.1306G > T, p.Glu436*	Nonsense	Unknown	7	1	70
49^†^	c.998delT, p.Leu333Trpfs*16	Frameshift	Unknown	21	0	N/A
25	c.2069G > A, p.Arg690His; c.G1650T, p.Lys550Asn	Missense Missense	Yes Unknown	49 28	1	40
22	c.1622G > A, p.Cys541Tyr; c.2048C > A, p.Thr683Asn	Missense Missense	Yes Unknown	37 45	1	80
33	c.619C > T, p.Arg207*; c.2051G > C, p.Arg684Pro	Nonsense Missense	Yes Unknown	54 36	2	40
40	c.187C > T, p.Arg63*; c.2196-2delA	Nonsense Splice	Yes Unknown	50 45	2	80
10	c.73C > T, p.Arg25Ter; c.632_635delAAAG, p.Glu211Alafs*29	Nonsense Frameshift	Yes Unknown	49 48	1	N/A

*IHC* immunohistochemistry, *N/A* not assessed, *VAF* variant allele frequency

^†^Patient with monosomy 7 or del(7q)

Both *EZH2*-mut AML and MDS/MPN patients had a median of 4 mutations (Fig. 1); range 1–7 in AML, and 3–7 in non-AML patients. In *EZH2*-mut AML patients, co-occurring mutations were most frequently found in *CEBPA* (4/6, 67%), *ASXL1* (3/6, 50%), *TET2,* and *RAD21* (2/6, 33% each). In *EZH2*-mut MDS/MPN patients, co-mutations were most common in *ASXL1* (7/7, 100%), *NRAS*, *RUNX1*, and *STAG2* (2/7, 29% each). None of the *EZH2*-mut patients had a mutation in *FLT3* or *NPM1*. In contrast, *EZH2*-wild-type (wt) AML patients had a median of 2 mutations, most frequently in *DNMT3A* (13/45, 29%), *NRAS* (10/45, 22%), *NPM1* (9/45, 20%), *FLT3, IDH1, TET2* (7/45 each, 16%), *ASXL1,* and *TP53* (6/45 each, 13%).

Regarding chromosome 7 abnormalities, 13/51 AML patients (25%) and 2/7 MDS/MPN patients (29%) had a deletion of the long arm, i.e. del(7q) or monosomy 7 (Fig. 1, Table 2, Additional File 1: Table S1). Deletion of the *EZH2* gene on chromosome 7q36 was confirmed by fluorescence in situ hybridization (FISH) in 2 out of 3 del(7q) samples (Additional File 1: Table S1). Notably, in the entire cohort the prevalence of *EZH2* mutations was similar in patients with del(7q)/-7 lesions (3/15, 20%) and with structurally normal chromosome 7 (10/43, 23%). In the AML cohort, chromosome 7 abnormalities were present in 27% of *EZH2*-wt and 17% of *EZH2*-mut patients.

### EZH2 expression is reduced in patients with mutated EZH2

**Fig. 2:**
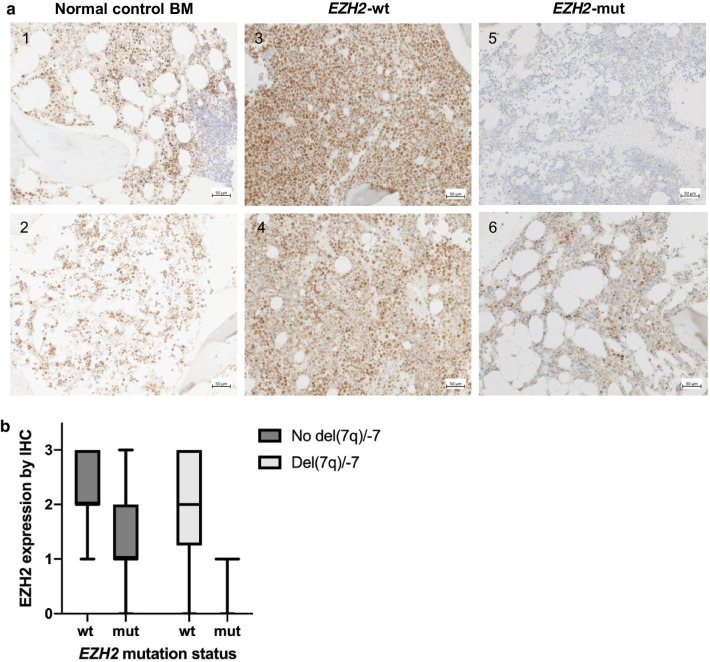
*EZH2* mutations result in decreased EZH2 protein expression. **a** Representative examples of EZH2 immunolabeling of control and four patient BM biopsies. Immunolabeling of normal BM biopsies shows on average a moderate nuclear expression level score of 2 in hematopoietic cells (samples 1, 2) while a reactive nodular lymphoid infiltrate in (1) is completely negative. BM samples of two *EZH2*-wt patients show strong (sample 3, IHC score 3) or moderate (sample 4, score 2) EZH2 expression. In contrast, EZH2 staining intensity is negative (sample 5, score 0) or weakly positive (sample 6, score 1) in BM biopsies of two *EZH2*-mut patients. Samples 3 and 5 are derived from patients with intact chromosome 7, samples 4 and 6 from patients with del(7q)/-7. **b** Median and range of EZH2 protein expression by IHC depending on *EZH2* mutation and chromosome 7 status. EZH2 expression is lower (*p* = 0.0002 by two-way ANOVA) in patients with an *EZH2* mutation compared to patients with wild-type *EZH2*. The reduction is similar in patients with and without chromosome 7q losses or deletions, but patients with del(7q)/-7 tended to have lower EZH2 expression levels than patients with intact chromosome 7 (*p* = 0.07)

Most *EZH2* mutations are predicted to result in loss of function of the protein, and loss of one *EZH2* allele through monosomy 7 or deletion of the long arm of chromosome 7 might decrease expression levels. To investigate whether EZH2 protein abundance could be decreased by these mechanisms, we evaluated EZH2 expression in bone marrow (BM) core biopsies from all 58 patients by employing immunohistochemistry (IHC). EZH2 expression levels in neoplastic cells ranged from no (score 0) to strong expression (score 3) while the hematopoietic BM cells of healthy donors exhibited moderate expression (score 2; Fig. 2a). In the entire cohort, the expression score was 0 in 3 patients, 1 in 13, 2 in 23, and 3 in 19 patients, respectively. Consequently, 16 patients were classified as having low (IHC score of 0–1) and 42 patients as having high EZH2 expression (IHC score 2–3).

We next asked whether EZH2 protein expression differs between patients depending on *EZH2* mutations and chromosome 7 abnormalities. EZH2 expression was lower in *EZH2*-mut patients than in *EZH2*-wt patients (*p* = 0.0002). The reduction in EZH2 expression was similar in patients with intact chromosome 7 and with del(7q)/-7, but EZH2 expression levels tended to be lower in patients with del(7q)/-7 than in patients with intact chromosome 7 (*p* = 0.07; Fig. 2b).

Median EZH2 expression in the 13 patients with mutations of this gene did not differ between de novo versus t-AML/AML-MRC, nor between AML and MDS/MPN. The presence of one or two *EZH2* mutations did not have a different impact on EZH2 expression (*p* = 0.81 by unpaired t-test) and no correlation was observed between the VAF of *EZH2* mutations and EZH2 expression (*R*^2^ = 0.02, *p* = 0.63).

### EZH2 expression and trimethylation of histone H3K27

**Fig. 3:**
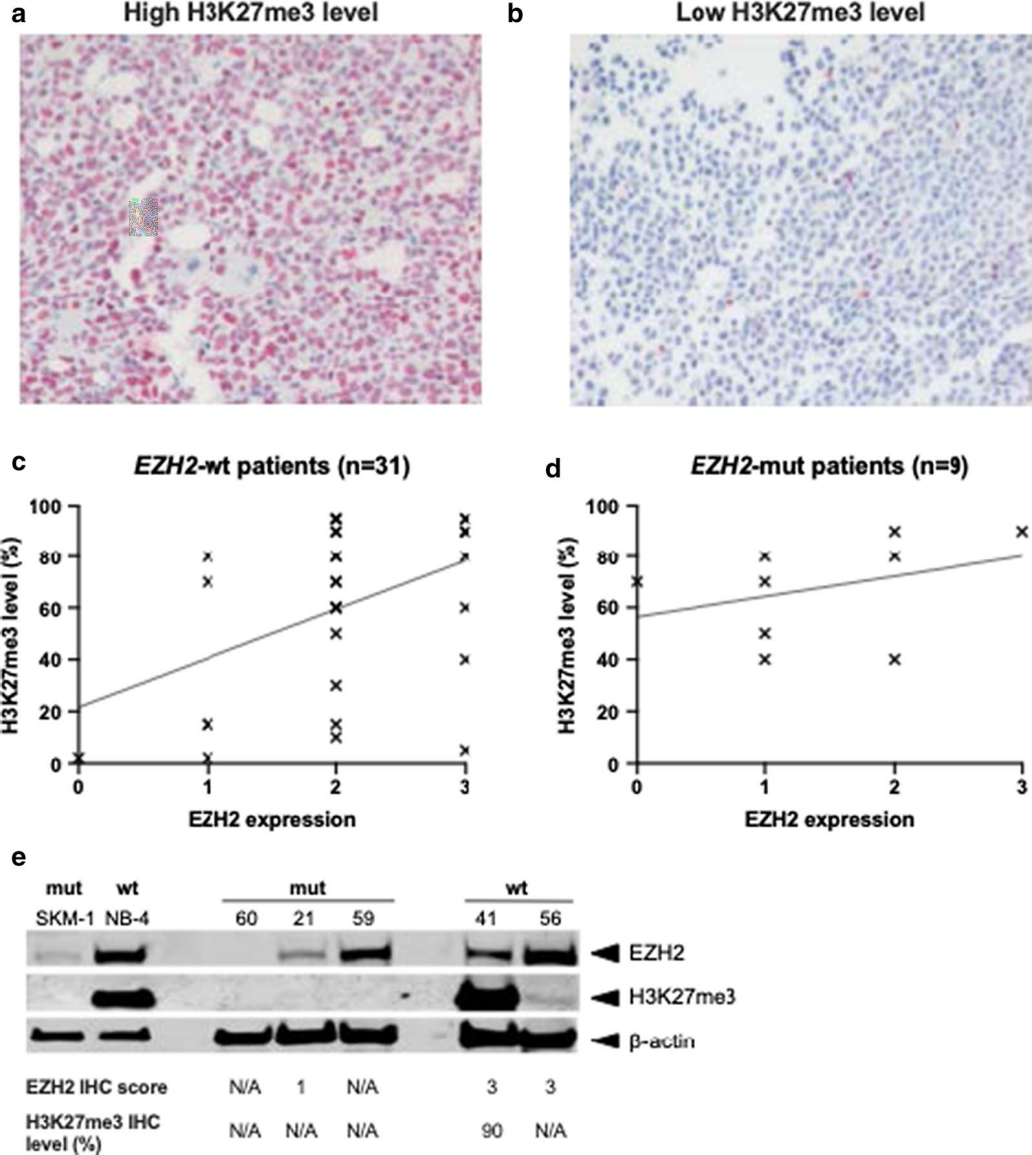
EZH2 expression and H3K27 trimethylation. **a** Representative example of strongly positive H3K27me3 expression in BM cells from a patient. **b** Representative example of low H3K27me3 expression in BM cells from a control case. **c** Linear regression analysis of 31 *EZH2*-wt cases shows an association between EZH2 expression and H3K27me3 levels, even though H3K27me3 levels are highly variable (*R*^2^ = 0.2, *p* = 0.01). **d** In 9 *EZH2*-mut cases, no association between EZH2 expression and H3K27me3 levels could be detected by linear regression analysis (*R*^2^ = 0.12, *p* = 0.36). **e** Western blot on *EZH2* mutant and wild-type AML cell lines and AML patient samples. Western blot results for EZH2 and H3K27me3 using whole cell extracts from *EZH2* mutant samples (mut; AML cell line SKM-1 and three AML patient samples) and *EZH2* wild-type samples (wt; APL cell line NB-4 and two AML patient samples). All patient data can be found in Tables 2 and 3. Beta-actin was used as loading control. Space between bands was cropped to save space

Since functional EZH2 protein is necessary for the trimethylation of H3K27, the presence of this histone mark was also determined by IHC in 40 patients, 9 of whom were *EZH2*-mut and 31 were *EZH2*-wt, with available BM biopsy material. The intensity of nuclear H3K27 staining in BM cells from individual cases was highly variable (Fig. 3a, b). Linear regression analysis showed an association between EZH2 protein expression (as assessed by IHC) and H3K27me3 levels in *EZH2*-wt patients, even though there was a high variability in H3K27me3 levels (*R*^2^ = 0.2, *p* = 0.01; Fig. 3c). For the 9 *EZH2*-mut cases, no correlation between EZH2 expression and H3K27me3 levels could be detected (*R*^2^ = 0.12, *p* = 0.36; Fig. 3d). The presence of one or two *EZH2* mutations did not have a different impact on H3K27 trimethylation (*p* = 0.32 by unpaired *t* test): median H3K27 trimethylation levels were 70% and 60% in patients with one or two *EZH2* mutations, respectively. However, this analysis was limited by the fact that H3K27 trimethylation levels were unavailable in 4 *EZH2*-mut patients (1 with one *EZH2* mutation and 3 with two *EZH2* mutations).

In order to confirm the observed variability of EZH2 expression and H3K27me3 levels using a second detection method, we also conducted immunoblotting. The AML cell lines selected were SKM-1 (*EZH2*-mut, carrying a homozygous A(1937) > G transition, resulting in a Y646C amino acid replacement) and NB-4 (*EZH2*-wt). The primary blasts were from 5 AML patients (3 *EZH2*-mut and 2 *EZH2*-wt). Neither of the cell lines (data from DSMZ) and none of the patients analyzed had loss of an *EZH2* allele (NB-4 cells carry a trisomy of chromosome 7). In our hands, SKM-1 cells did not disclose appreciable H3K27 trimethylation, which is in line with only low H3K27me3 levels described by Ernst et al. [6], and in striking contrast to NB-4 (Fig. 3e). *EZH2*-mut patient samples disclosed variable amounts of EZH2 protein: the respective band was not detectable in patient 60 (harboring an *EZH2* stop mutation at a VAF of 90%, no EZH2 IHC score available), reduced in patient 21 (two *EZH2* mutations with 9/11% VAF, EZH2 IHC score of 1), and abundant in patient 59 (*EZH2* frameshift mutation at a VAF of 78%, no EZH2 IHC score available). In the two *EZH2*-wt patients, EZH2 could readily be detected.

H3K27me3 levels were also quite variable, supporting the wide range observed across the 40 patients where BM biopsies were available for H3K27me3 IHC. Notably, H3K27me3 was absent in all 3 *EZH2*-mut patient samples, and present in the two *EZH2*-wt patients (albeit with reproducibly lower levels for patient 56 than for patient 41).

### AML patients with an EZH2 mutation or decreased EZH2 expression have inferior clinical outcome

More than half of the AML patients received induction chemotherapy and allogeneic hematopoietic stem cell transplantation (HSCT; Table 1). One quarter received non-intensive treatment regimens (mainly DNA-hypomethylating agents or sole best supportive care, with or without hydroxyurea). Information on treatment was missing for 1 patient in the AML cohort. In the MDS/MPN cohort, 4/7 patients received HSCT (none received induction chemotherapy) and 3/7 received non-intensive therapy (mostly hypomethylating agents).

Patients with AML with recurrent genetic abnormalities, or AML not otherwise specified (NOS), received induction chemotherapy more often than patients with AML-MRC or t-AML (84% versus 38%), while the frequency of HSCT was similar (52% and 54%, respectively). Non-intensive therapies were much less frequent in patients with AML with recurrent genetic abnormalities or AML NOS compared to patients with AML-MRC or t-AML (8% versus 46%).

To perform an exploratory and retrospective analysis of *EZH2* mutation status and EZH2 protein expression as parameters that might be associated with outcome, overall survival (OS) was determined from the time of diagnosis until death or last follow-up. Of the 58 patients, 34 have died, 24 were alive at last follow-up, and no patient was lost to follow-up, with a median follow-up time of 3.41 years.

**Fig. 4:**
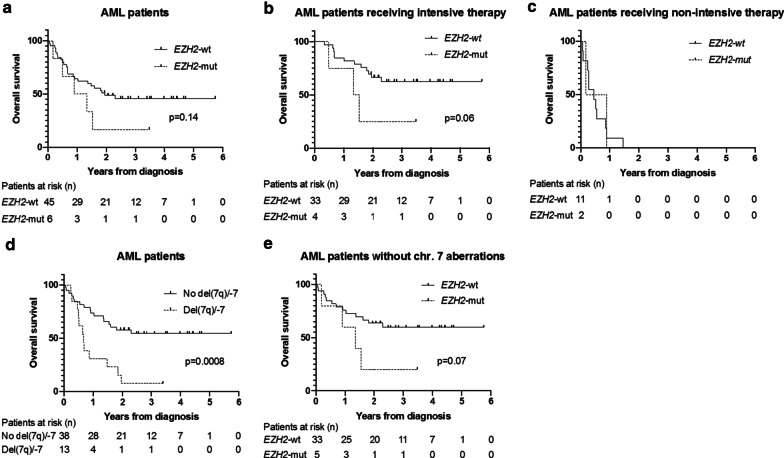
*EZH2* mutations and chromosome 7 aberrations are associated with inferior OS in AML patients. **a** OS of AML patients by *EZH2* mutation status. 21 patients in the *EZH2*-wt and one patient in the *EZH2*-mut group were censored (still alive at last follow-up). **b** The presence of an *EZH2* mutation is associated with inferior OS in the subgroup of AML patients having received intensive therapy. 21 patients in the *EZH2*-wt and one patient in the *EZH2*-mut group were censored (still alive at last follow-up). **c** In AML patients having received non-intensive therapy, OS is similar in *EZH2*-mut and *EZH2*-wt patients. **d** OS of AML patients by chromosome 7 status. 21 patients without chromosome 7 abnormalities and one patient with del(7q)/-7 were censored (still alive at last follow-up). **e** OS of AML patients by *EZH2* mutation status excluding patients with chromosome (chr.) 7 aberrations. 20 patients in the *EZH2*-wt and one patient in the *EZH2*-mut group were censored (still alive at last follow-up)

In the cohort of AML patients, median OS of *EZH2*-mut patients was 1.12 years versus 1.95 years in *EZH2*-wt patients (*p* = 0.14, Fig. 4a). Of note, *EZH2*-mut patients were older than *EZH2*-wt patients (median age of 71 versus 61 years), and 67% received induction chemotherapy and/or HSCT compared to 75% of *EZH2*-wt patients. Taking into account other known prognostically relevant factors, we conducted subgroup analyses with respect to treatment intensity, and the presence or absence of chromosome 7 aberrations by employing Kaplan–Meier plots. For the subgroup of AML patients having received intensive therapy (*n* = 37), the results were similar to those obtained in the entire AML cohort (Fig. 4b). Non-intensively treated AML patients, however, had a dismal outcome, irrespective of *EZH2* mutation status (Fig. 4c).

As expected, AML patients with chromosome 7 abnormalities had shorter OS than patients with intact chromosome 7 (median OS of 0.67 years versus not reached, *p* = 0.0008, Fig. 4d). In the subgroup of AML patients without chromosome 7 aberrations, OS was shorter in *EZH2*-mut than in *EZH2*-wt patients (median OS of 1.34 years versus not reached, *p* = 0.07, Fig. 4e).

**Fig. 5:**
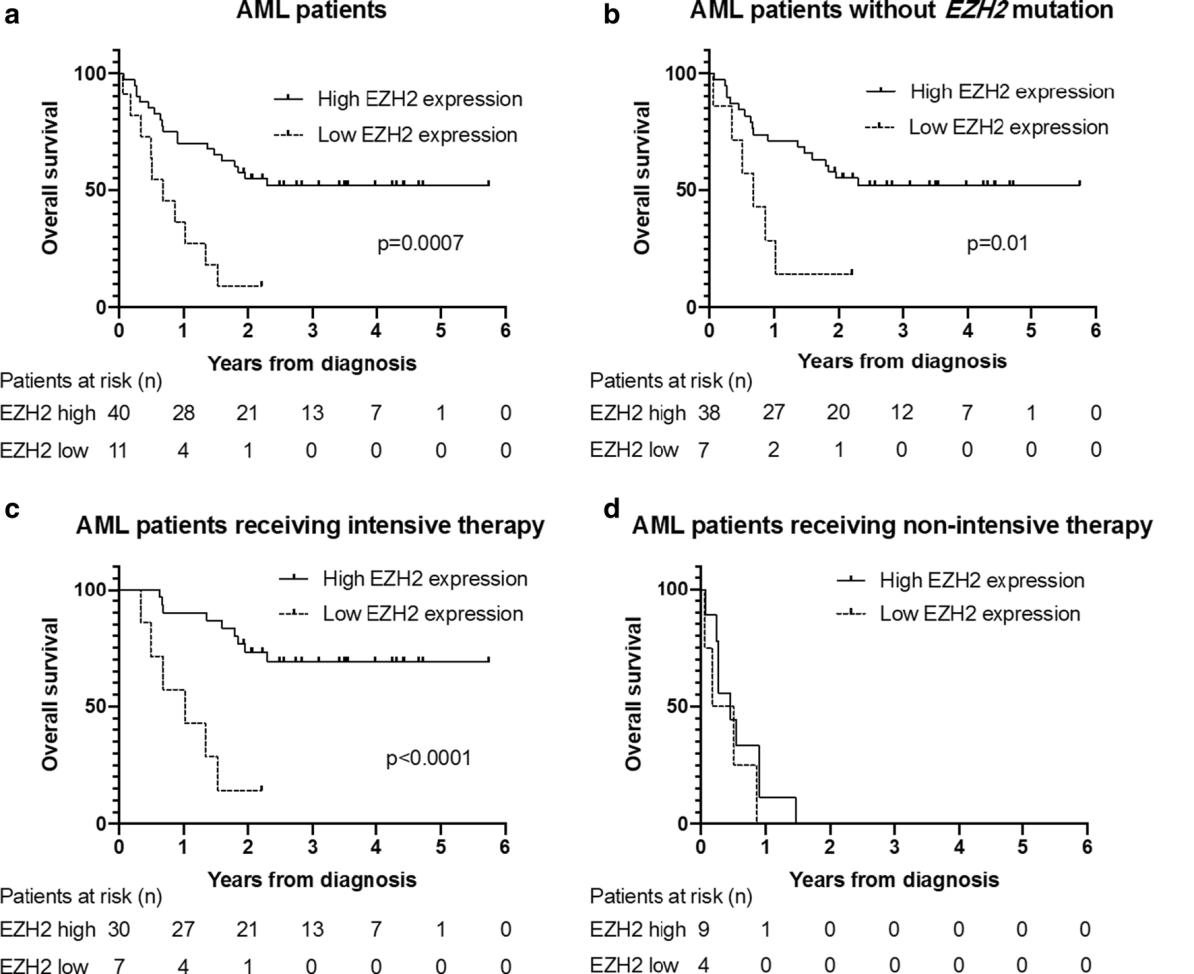
Decreased EZH2 protein expression is associated with inferior OS in AML patients. **a** OS of AML patients by EZH2 protein expression level. 21 patients in the EZH2-high and one patient in the EZH2-low group were censored (still alive at last follow-up). **b** OS of AML patients by EZH2 protein expression excluding *EZH2*-mutated patients. 20 patients in the EZH2-high and one patient in the EZH2-low group were censored (still alive at last follow-up). **c** Low EZH2 protein expression is associated with inferior OS in the subgroup of AML patients having received intensive therapy. 21 patients in the EZH2 high and one patient in the EZH2 low group were censored (still alive at last follow-up). **d** In AML patients having received non-intensive therapy, OS is similar in patients with high or low EZH2 expression

Next, we evaluated the prognostic impact of EZH2 protein expression in BM cells. In the cohort of AML patients, OS was significantly shorter in patients with low EZH2 expression (IHC score of 0–1) than in patients with high EZH2 expression (IHC score of 2–3; median OS of 0.68 years versus not reached, *p* = 0.0007, Fig. 5a). This difference in OS was maintained when excluding the 6 *EZH2*-mut AML patients from the analysis (Fig. 5b). For AML patients having received intensive therapy (n = 37), the association of EZH2 expression with OS was even more pronounced (Fig. 5c). In AML patients having received only non-intensive therapy, however, no association between EZH2 expression and OS was observed (Fig. 5d). Similar results were obtained when looking at OS according to EZH2 expression in the entire cohort of intensively or non-intensively treated AML and MDS/MPN patients (Additional File 2: Figure S1A, B).

After multivariable adjustment, considering the type of treatment (intensive or non-intensive), the disease (AML or non-AML myeloid neoplasm), and the presence of del(7q)/-7, the presence of an *EZH2* mutation was associated with a trend towards an increased risk of death (hazard ratio (HR) 2.51 [95% confidence interval (CI) 0.87–7.25], *p* = 0.09); similarly, low EZH2 protein expression was associated with elevated risk (HR 2.54 [95% CI 1.07–6.04], *p* = 0.04).

### Chromosomal loss of one EZH2 allele does not attract aberrant DNA hypermethylation of the remaining allele at the 5′ region of the EZH2 gene

**Fig. 6:**
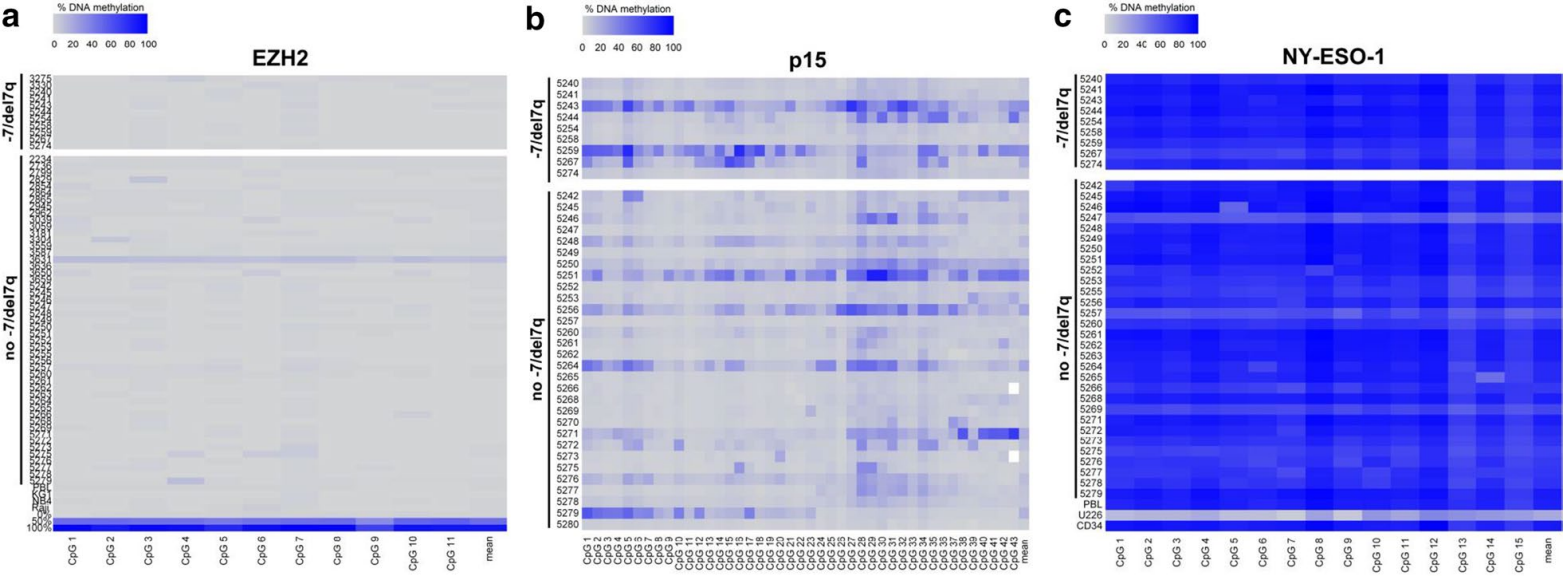
Chromosome 7 abnormalities do not affect the methylation status of the *EZH2* promoter. DNA methylation levels were determined by bisulfite pyrosequencing in a cohort of MDS/low blast count AML patients. Four-digit numbers represent DNA patient samples. Continuous color gradients represent methylation values between 0% (grey) and 100% (blue). Mean DNA methylation is indicated for each sample. **a** DNA hypomethylation of the *EZH2* promoter in MDS/AML patients. DNA methylation levels of 11 CpGs located within the *EZH2* promoter of 60 patients with (*n* = 11) or without (*n* = 49) del(7q)/-7 are shown. Peripheral blood lymphocytes (PBL) from a healthy control and the cell lines KG-1, NB-4, and Raji, as well as 0%, 50%, and 100% in vitro methylated DNA served as controls. **b** DNA methylation of the *p15/INK4B* (*CDKN2B*) promoter of MDS patients. DNA methylation levels of 43 CpGs located within the *p15/INK4B* promoter of 39 patients with (*n* = 9) or without (*n* = 30) del(7q)/-7 are shown. **c** DNA hypermethylation of the *NY-ESO-1* promoter of MDS patients. DNA methylation levels of 15 CpGs located within the *NY-ESO-1* promoter of 39 patients with (*n* = 9) or without (*n* = 30) del(7q)/-7 are shown. PBL from a healthy control, the human myeloma cell line U226, and CD34 + stem cells served as controls

Aberrant DNA hypermethylation of tumor suppressor genes constitutes a very frequent epigenetic mechanism of gene repression. There is first evidence that this mechanism may be particularly active on genes residing on residual chromosomes that persist after the other copy has been lost, e.g. with a monosomy 7 state [18, 19]. Since chromosome 7 abnormalities are frequent in AML and MDS, we asked whether the CpG island of the *EZH2* promoter would attract aberrant DNA hypermethylation in the mono-allelic but not the normal, diploid state. Therefore, BM mononuclear cells from a cohort of 62 patients (Table 4), 57 with MDS or MDS/MPN, 5 with AML, 11 of them with del(7q)/-7, and 51 without these chromosome 7 abnormalities, were subjected to quantitative methylation analysis at 11 CpGs in this region. All CpGs had low levels of methylation (< 5%), and there was no difference between the 2 cytogenetic groups, also when patients were serially studied, i.e. before and after decitabine (DAC) treatment (Fig. 6a, Additional File 2: Figure S2).

**Table 4:** Patient characteristics of the MDS/low blast count AML cohort subjected to methylation analyses

Number of patients	62
Age (years; median and range)	70.5 (20–83)
Sex	
Male	40
Female	22
FAB subtype	
RA	17
RARS	2
RAEB	35
RAEB-t	5
CMML	3
IPSS risk category	
Low	5
Intermediate I	17
Intermediate II	30
High	10
Del(7q) or -7 (%)	11 (18)

*CMML* chronic myelomonocytic leukemia, *IPSS* International Prognostic Scoring System, *RA* refractory anemia, *RAEB* refractory anemia with blast excess, *RAEB-t* refractory anemia with blast excess in transformation (i.e., acute myeloid leukemia according to the World Health Organization classification), *RARS* refractory anemia with ring sideroblasts

We also performed control experiments by profiling 43 CpGs from the CpG island of the *p15/INK4B* (*CDKN2B*) promoter (located on chromosome 9p21), known to be variably methylated in AML/MDS [20], and of the CpG island of the *NY-ESO-1* promoter (located on the X chromosome) known to be very heavily methylated in primary AML/MDS [21]. For these genes, no difference in overall methylation could be observed between the 2 cytogenetic subgroups, either (Fig. 6b, c).

## Discussion

Multiple genes located in chromosomal regions frequently deleted in myeloid malignancies (i.e. the long arms of chromosome 7 and 5, and the short arm of chromosome 17) become monoallelic when such deletions occur. Thus, it is not surprising that for *EZH2*—central in maintaining PRC2 activity—tumor suppressor function has been proposed when in 2010, inactivating point mutations in myeloid neoplasms were discovered by different groups [6–8]. Since then, the prevalence of *EZH2* mutations, their association with deletions of the other allele, their pathogenic role [22], and negative clinical implications [9, 23] have been broadly studied in MDS/MPN cohorts where alterations of this gene are relatively frequent and often associated with mutations in *ASXL1* (also a member of the Polycomb group of proteins), *TET2*, and *RUNX1* [24]. In AML, *EZH2* mutations have been reported with a prevalence of about 2% in more than 1500 patients comprehensively profiled for recurrent mutations [12], and to be highly specific for secondary AML [25].

In the present study, we confirmed and extended previous investigations describing missense and nonsense mutations in AML and MDS. Compared to the results of others looking at different AML subtypes in a much larger cohort of patients [25], we also found that AML patients harboring *EZH2* mutations were mostly older individuals and had a median of 4 mutations in genes associated with myeloid malignancies. The *EZH2* mutation rate of 9% in AML-MRC matches the one previously reported for secondary AML. In contrast, the *EZH2*-mutation rates in de novo AML and t-AML in our cohort are higher than those previously reported, which is probably due to the difference in sample size.

Complementing the mutational status with associated recurrent genetic alterations, we also noted the frequent co-occurrence of *EZH2* mutations with mutations in *ASXL1* (also resulting in reduced PRC2 function), *TET2,* and *RUNX1*. In contrast, in patients with *EZH2* in wild-type configuration, mutations in *DNMT3A*, *NRAS*, *NPM1,* and *FLT3* were most frequent. Regarding concomitant mutations and simultaneous loss of the second *EZH2* allele, the prevalence of both lesions as compared to either mutation or chromosomal loss was not as pronounced as has been described e.g. for *TP53* in AML [12], where Knudson's two-hit hypothesis of sequential tumor suppressor gene inactivation fully bears out. These results are thus in line with those described by Bejar et al. [9].

Regarding the impact of altered *EZH2* status on EZH2 protein expression, markedly reduced protein abundance was observed in patients with inactivating mutations and, to a lower extent, those with chromosomal loss of one copy of the gene. The reduced EZH2 protein expression in mutant compared to wild-type samples is in line with results published by McGraw et al. [23]. However, in their cohort of myelodysplasia-related neoplasms, del(7q)/-7 lesions were overrepresented in *EZH2*-mutated cases, resulting in a significantly lower EZH2 protein expression in del(7q)/-7 cases compared to cases with intact chromosome 7. Our results suggest that overexpression e.g. from increased transcription of the remaining (unmutated) copy of *EZH2* may represent a compensatory mechanism.

Given the central role of EZH2 for trimethylation of H3K27, it was of interest to ask whether an association exists between lower EZH2 levels and decreased abundance of this "silencing" mark, as previously reported e.g. by Göllner et al. [4]. A significant relationship between EZH2 protein expression and H3K27me3 levels could be observed only in *EZH2*-unmutated patients, even though H3K27me3 levels showed a heterogeneous distribution. In *EZH2*-mutated patients no correlation could be detected; however, the sample size was limited.

Aberrant promoter hypermethylation can silence the transcriptional activity of tumor suppressor genes. It has been hypothesized that chromosomal loss resulting in heterozygosity and decreased gene dosage of tumor suppressor genes on the long arms of chromosome 5 or 7, may attract additional, epigenetic gene silencing [18, 19]. We tested this hypothesis for the *EZH2* gene by determining the methylation status of 11 CpGs located in the promoter region by pyrosequencing in 11 MDS/AML patients with loss of one *EZH2* allele and 49 patients that were diploid for this gene by cytogenetics or FISH analyses. This gene region was uniformly unmethylated in both cytogenetic groups, thus not attracting aberrant hypermethylation when monoallelic in this patient cohort. As the *EZH2* promoter was already hypomethylated prior to therapy, no additional, decitabine-induced hypomethylation responses could occur.

Focusing on the impact of *EZH2* mutations and decreased protein expression on clinical outcome, we confirmed the previously reported impaired OS observed in patients with *EZH2* mutations and decreased EZH2 protein expression [4, 9, 23]. The prognostic impact of EZH2 alterations is influenced by the disease context and treatment, as shown in our multivariable and subgroup analyses.

Although our analyses are limited by the sample size, these results suggest that EZH2 expression may add to the predictive value of the mutational spectrum routinely interrogated in AML patients by next-generation sequencing (NGS). Mechanistically, it would be of great interest to interrogate differential H3K27me3 marking at specific gene loci which may be implicated in the aggressiveness of different myeloid malignancies.

## Conclusions

*EZH2* mutations in AML/MDS patients are associated with decreased EZH2 protein expression. The mutation of one *EZH2* allele is more effective in reducing EZH2 expression than loss of one allele through loss of chromosome 7 or 7q. Chromosomal loss of one *EZH2* allele does not result in *EZH2* promoter hypermethylation. EZH2 mutations and decreased protein expression are associated with inferior survival. The evaluation of EZH2 expression might favorably complement the routine molecular profiling of patients with myeloid neoplasms.

## Patients and methods

We studied 58 patients (51 AML, 3 MDS, 3 MDS/MPN, 1 MPN; Table 1) from a single center, almost all diagnosed between 2015 and 2017. AML cases were selected for having available NGS data, and BM biopsies adequate for immunohistochemical analyses. Non-AML patients were further selected for harboring an *EZH2* mutation. Two additional *EZH2*-mut AML cases (having received non-intensive treatment) were included exclusively for western blot analysis. All patients had provided written informed consent to BM studies according to institutional standards.

### Genetic analyses

Mutation status was determined by NGS targeting a panel of 54 genes (Illumina TruSight Myeloid Sequencing Panel, San Diego, CA, USA). Standard metaphase cytogenetics and/or FISH and single nucleotide polymorphism arrays (CytoScan HD arrays and assays in selected patients) were conducted to determine *EZH2* copy number status.

### Protein expression studies

#### Immunohistochemistry

Protein expression of EZH2 and H3K27me3 in primary patient cells and normal BM cells from healthy donors was assessed by IHC on formalin-fixed, EDTA-decalcified, paraffin-embedded BM core biopsies. The Ezh2 (AC22) mouse monoclonal antibody #3147 (Cell Signaling Technology, Danvers, MA, USA) and the histone H3K27me3 rabbit polyclonal antibody #39,155 (Active Motif, Carlsbad, CA, USA) were used for IHC. For EZH2 protein expression analyses, 12 control age- and sex-matched BM biopsy samples were retrieved from the archives. The biopsies had originally been performed on healthy hematopoietic stem cell donors or lymphoma patients during the staging procedure. EZH2 protein status was scored independently, and in a blinded fashion, by two trained investigators (ASG, RdL). Intensity of EZH2 protein immunolabeling was assessed as 0 (negative), 1 + (weakly positive), 2 + (moderately positive), or 3 + (strongly positive). Semiquantitative evaluation of staining intensity was performed according to the average color rendering degree by bright field microscopy. Absence of nuclear EZH2 expression or faint nuclear staining was recorded as 0; weak but distinct expression higher than the background was recorded as 1; moderate color rendering significantly higher than the background was recorded as 2; strong expression corresponding to a dark brown nuclear immunolabeling was scored as 3.

As for H3K27me3, the intensity of nuclear staining in BM cells from individual cases was highly variable. Therefore, no intensity scoring as in the evaluation of EZH2 protein expression could be performed. Instead, we evaluated the percentage of positive nuclei including various staining intensities (Fig. 3a, b).

#### Fluorescent western blot

Whole cell extracts from cultured cell lines and patient samples were used to perform western blots. LDS sample buffer and Reducing Agent (Novex) was added, samples were heated for 5–10 min at 90 °C, loaded onto 4–12% SDS–polyacrylamide gel (Novex) and electrophoresed at 180 V for 30 min in MES SDS running buffer (Novex). Transfer was performed using a XCell SureLock Electrophoresis Cell at 30 V for 90 min using PVDF membrane (Millipore) and transfer buffer (Novex, 20% methanol, 1% antioxidant). The membrane was blocked in blocking buffer (5% bovine serum albumin (BSA) in TBS-T (Tris-buffered saline with 0,1% Tween-20, pH 7.6.) for 60 min, shaking at room temperature. Antibodies were used at the following dilutions: EZH2 1:2500, beta-actin 1:320,000, H3 and H3K27me3 1:1000 in 5% BSA TBS-T and the membrane was incubated overnight rocking at 4 °C. The next day, the membrane was washed three times for 10 min in TBS-T. Multiplexed IRDye secondary antibodies (LI-COR) were used at a dilution of 1:20,000 in 5% BSA TBS-T, and the membrane was incubated for 60 min at room temperature. After washing with TBS-T, the signals were detected using the 700 nm and 800 nm channels of the Odyssey CLx imaging system operated by the Image Studio software (LI-COR). A list of antibodies used in this study is provided in Additional File 1: Table S2.

### DNA methylation analysis

DNA methylation levels were determined by bisulfite pyrosequencing on mononuclear BM cells, isolated by Ficoll gradient sedimentation, in 62 MDS/AML patients (Table 4). 500 ng of genomic DNA was sodium bisulfite-modified using the EZ DNA Methylation Kit (Zymo Research, Irvine, CA, USA). Quantitative DNA methylation was assessed by pyrosequencing as previously described [26] using the PyroMark Q96 MD system (Qiagen, Hilden, Germany). 11 CpGs located within a 113 base pair region of the *EZH2* promoter, 43 CpGs within the *p15/INK4b* (*CDKN2B)* promoter, and 15 CpGs within the *NY-ESO-1* promoter were analyzed for methylation (primer sequences provided in Additional File 1: Table S3). Peripheral blood lymphocytes from a healthy donor, CD34 + hematopoietic progenitor cells and cell lines (KG-1, NB-4, Raji, U226) were used as controls. To control for PCR bias, 0%, 50%, and 100% DNA methylation standards were obtained by mixing unmethylated and 100% in vitro methylated DNA (M. Sssl, New England Biolabs, MA, USA) at defined ratios.

### Statistical analyses

Two-way ANOVA and Tukey’s multiple comparisons test were used to assess whether EZH2 protein expression was associated with *EZH2* mutation and copy number status. Linear regression analysis was used to assess a potential association between EZH2 protein expression and H3K27me3 levels.

OS was determined from the time of diagnosis until death or last follow-up. Survival curves were calculated using the Kaplan–Meier method and compared using the Log-rank test. Cox proportional hazard regression was used to investigate the association between EZH2 mutational status or protein expression and OS, adjusted for relevant known prognostic factors (treatment, disease, del(7q)/-7). Statistical analyses were performed with GraphPad Prism V8.3 (San Diego, CA, USA) and SAS V9.2 (SAS Institute Inc., Cary, NC, USA).

## Supplementary information

**Additional file 1**. Supplementary Tables.

**Additional file 2**. Supplementary Figures.

## Data Availability

The datasets used and/or analysed during the current study are available from the corresponding author on reasonable request.

## Abbreviations

-7: monosomy 7
AML: acute myeloid leukemia
APL: acute promyelocytic leukemia
ATRA: all-*trans* retinoic acid
AZA: azacitidine
BSA: bovine serum albumin
BM: bone marrow
Chr.: chromosome
CI: confidence interval
CMML: chronic myelomonocytic leukemia
CNL: chronic neutrophilic leukemia
DAC: decitabine
Del(7q): 7Q deletion
DSMZ: German collection of microorganisms and cell cultures
EB: excess blasts
EZH2: enhancer of zeste homolog 2
FISH: fluorescence in situ hybridization
H3K27me3: H3K27 trimethylation
HR: hazard ratio
HSCT: hematopoietic stem cell transplantation
HU: hydroxyurea
IHC: immunohistochemistry
LDAC: low dose cytarabine
MDS: myelodysplastic syndrome
MLD: multilineage dysplasia
MPN: myeloproliferative neoplasm
MRC: myelodysplasia-related changes
Mut: mutated
N/A: not assessed
NGS: next-generation sequencing
NOS: not otherwise specified
OS: overall survival
PB: peripheral blood
PBL: peripheral blood lymphocytes
PRC2: polycomb repressive complex 2
t-AML: therapy-related AML
TBS: tris-buffered saline
VAF: variant allele frequency
WBC: white blood cells
Wt: wild-type
