# Correction to: Integrative study of EZH2 mutational status, copy number, protein expression and H3K27 trimethylation in AML/MDS patients

**DOI:** 10.1186/s13148-021-01087-5

**Published:** 2021-04-28

**Authors:** Julia Stomper, Ruth Meier, Tobias Ma, Dietmar Pfeifer, Gabriele Ihorst, Nadja Blagitko-Dorfs, Gabriele Greve, Dennis Zimmer, Uwe Platzbecker, Anne Hagemeijer, Ingrid Schmitt-Graeff, Michael Lübbert

**Affiliations:** 1 Department of Medicine I (Hematology, Oncology and Stem Cell Transplantation), Medical Center – University of Freiburg, Freiburg, Germany; 2 Clinical Trials Unit, Faculty of Medicine, Medical Center - University of Freiburg, Freiburg, Germany; 3 Institute for Immunodeficiency, Center for Chronic Immunodeficiency (CCI), Medical Center - University of Freiburg, Freiburg, Germany; 4 German Cancer Consortium (DKTK) and German Cancer Research Center (DKFZ), Partner Site Dresden, Dresden, Germany; 5 Medical Department-Hematology and Cell Therapy, Medical Oncology, Hemostaseology, University of Leipzig Medical Center, Leipzig, Germany; 6 University of Leuven, Leuven, Belgium; 7 University of Freiburg, Freiburg, Germany; 8 German Cancer Consortium (DKTK) and German Cancer Research Center (DKFZ), Partner Site Freiburg, Freiburg, Germany; 9 Faculty of Medicine, University of Freiburg, Freiburg, Germany

## Correction to: Clin Epigenet (2021) 13:77 10.1186/s13148-021-01052-2

After publication of the original article [1], the authors identified an error in the author name of Schmitt-Graeff.

The incorrect author’s name is: Annette Schmitt-Gräf.

The correct author’s name is: Ingrid Schmitt-Graeff.

The author group has been updated above and the original article [1] has been corrected.
