# Supplementary material for: Integrative study of EZH2 mutational status, copy number, protein expression and H3K27 trimethylation in AML/MDS patients
Source: Clin Epigenetics. 2021 Apr 12;13:77. doi: 10.1186/s13148-021-01052-2 (PMC8043064; doi:10.1186/s13148-021-01052-2)
Supplement: Supplementary file 1 — Additional file 1. Supplementary Tables. [file 13148_2021_1052_MOESM1_ESM.docx]

**Supplementary Information**

**Supplementary Table 1: Clinical characteristics of *EZH2*-unmutated AML patients**

| **Patient #** | **Sex** | **Age (years)** | **Disease subtype** | **Karyotype** | **Del(7q) or -7** | **% monosomy 7 cells (FISH)** | **WBC (x10^9^/L)** | **PB blasts (%)** | **Treatment** |
| --- | --- | --- | --- | --- | --- | --- | --- | --- | --- |
| *Patients with chromosome 7 aberrations* | | | | | | | | | |
| 6 | f | 46 | AML-MRC | 45,XX,-7,der(11)t(1;11)(q21;q23) [5]/46,XX [1] | Yes | 2 | 2.09 | 3 | Induction chemo, HSCT |
| 32 | f | 77 | AML-MRC | 45,XX,add(4)(p11),del(5)(q13q33),-7,-17, del(20)(q11),hsr(22)(q11),+r [5]/46,XX,idem,-4,-16,-18,add(21)(p11),+1-2mar [15]/46,XX [3] | Yes† | 20 | 2.44 | 17 | DAC, HSCT |
| 47 | m | 72 | AML-MRC | 46,XY,inv(3)(q21q26.2) [21] | Yes | 20 | 128.28 | 38 | HU |
|  |  |  |  | 45,XY,-7 (20%)*/+15(q22) (60%)* |  |  |  |  |  |
| 3 | f | 50 | AML-MRC | 46,XX,del(7)(q22q32) [9]/46,idem,del(13)(q14q22) [5]/47,XX,+8 [2]/46,XX, -3,del(6)(q13),  dic(3;14)(p13;q32),+14 [4]/46,XX [7] | Yes† | 26 | 0.71 | 25 | Induction chemo, HSCT |
| 4 | m | 79 | AML-MRC | 45,XY,-7 (33%)* | Yes | 33 | 3.46 | 28 | DAC, ATRA, VPA |
| 1 | m | 86 | AML-MRC | 44,XY,-3,-4,der(5)del(5)(p13p15)del(5)(q21q35),  -7,+8,der(11)(3qter->3q21::4q35->4q13::11p15  ->11qter) [8]/46,XY [4] | Yes | 52 | 1.81 | 2 | DAC, LDAC, tranylcypromine, ATRA, low dose melphalan |
| 26 | f | 61 | t-AML | 46,XX,del(5q31/5q33)del(7q22/7q31)del(12p13) (60%)*/47,XX,+11 (40%)* | Yes† | 78 | 2.33 | 45 | Induction chemo, HSCT |
| 17 | m | 70 | AML-MRC (history of CMML) | 46,XY,t(2;3)(p21;q26) [20]  45,XY,-7,del(16q22),i(17q) (80%)* | Yes | 80 | 98.46 | 33 | Induction chemo, DAC, HSCT |
| 30 | m | 67 | AML-MRC | 46,XY,del(7)(q22q32) [17]/46,XY [3] | Yes | 88 | 3.14 | 0 | AZA, HU |
| 13 | f | 76 | AML-MRC | ~44-45,XX,der(2)add(2)(p21)t(2;13)(q33;q14),  -5,add(7)(q22),+8,der(11)add(11)(p15)hsr(11)(q23)  add(11)(q23),-13,-17,-19x2,+r,+3-5mar,dmin [13]/ ~44-45,idem,-4 [10] | Yes† | 90 | 28.99 | 73 | DAC, AZA, HU |
| 54 | f | 32 | AML-MRC | 45,XX,-7 [22]/46,XX [1] | Yes | 94 | 15.43 | 68 | Induction chemo, HSCT |
| 53 | f | 77 | AML-MRC | 45,XX,-7 [20] | Yes | 95 | 46.99 | 72 | DAC |
| *Patients without chromosome 7 aberrations* | | | | | | | | | |
| 2 | f | 73 | AML, NOS | 46,XX [20] | No | 0 | 3.0 | 87 | Induction chemo, HSCT |
| 7 | f | 68 | AML-MRC | 46,XX,del(5)(q22q34),del(12)(p12p13) [9]/47,idem,+8 [4]/46,XX [7] | No | 0 | 1.87 | 1 | Induction chemo, HSCT |
| 8 | f | 20 | AML-MRC | 46,XX [20] | No | 0 | 1.31 | 1 | Induction chemo, HSCT |
| 9 | f | 63 | t-AML | 46,XX [23] | No | 0 | 66.66 | 86 | DAC, HSCT |
| 11 | m | 41 | AML-MRC | 46,XY,add(13)(q32) [13]/47,idem,+8 [12] | No | 0 | 10.4 | 13 | Induction chemo, HSCT |
| 12 | f | 65 | t-AML | 46,XX,t(8;21)(q22;q22.1) [15]/45,idem,-X [5] | No | 0 | 17.9 | 29 | DAC, HSCT |
| 15 | m | 36 | AML with recurrent genetic abnormalities | 46,XY [20] | No | 0 | 53.59 | 78 | Induction chemo, HSCT |
| 16 | m | 72 | AML, NOS | 46,XY [20] | No | 0 | 25.07 | 6 | Induction chemo, HSCT |
| 18 | m | 45 | AML with recurrent genetic abnormalities | 46, XY,ish ins16(q22p13p13)16p13(MYH11+) 16q22(CBFB+,MYH11+) [16]/46,XY [4] | No | 0 | 67.18 | 70 | Induction chemo, dasatinib, HSCT |
| 19 | m | 55 | AML with recurrent genetic abnormalities | 46,XY [20] | No | 0 | 56.27 | 55 | Induction chemo, midostaurin, HSCT |
| 20 | f | 51 | AML with recurrent genetic abnormalities | 46,XX,t(8;21)(q22;q22) [6]/46,idem,del(2)(p21) [14] | No | 0 | 3.96 | 28 | Intensive chemo |
| 23 | m | 25 | AML with recurrent genetic abnormalities | 45,X,-Y,t(8;21)(q22;q22) [21] | No | 0 | 18.17 | 62 | Intensive chemo |
| 24 | f | 56 | AML-MRC | 46,XX,t(11;16)(q23;q12) [9]/46,XX [11]  nuc ish 11q23 (5`MLLx2)(5`MLL sep 3`MLLx1) (51%)* | No | 0 | 0.62 | N/A | DAC, LDAC, HU |
| 28 | m | 80 | AML-MRC | 44,XY,der(5)t(5;11)(q14;q13),-11, dic(15;20)(p11;q11),der(17)t(8;17)(q11;p13),-18,+mar,1~10dim [5]/43,idem,-8 [12]/46,XY [3] | No | 0 | 1.32 | 5 | No information |
| 29 | m | 22 | AML with recurrent genetic abnormalities | 46,XY [20] | No | 0 | 24.6 | 2 | Intensive chemo |
| 31 | f | 48 | AML with recurrent genetic abnormalities | 46,XX,inv(16)(p13.1q22) [22] | No | 0 | 9.21 | 18 | Intensive chemo |
| 34 | f | 42 | AML with recurrent genetic abnormalities | 46,XX,t(8;21)(q22;q22) [13]/47,idem,+4 [11] | No | 0 | 11.05 | 66 | Intensive chemo, dasatinib |
| 36 | f | 77 | AML-MRC | 46,XX [20] | No | 0 | 2.73 | 0 | LDAC, tranylcypromine, ATRA |
| 37 | f | 41 | AML with recurrent genetic abnormalities | 46,XX [12] | No | 0 | 27.8 | 71 | Induction chemo, midostaurin, HSCT |
| 38 | f | 61 | AML with recurrent genetic abnormalities | 47,XX,+8 [2]/46,XX [21] | No | 0 | 2.34 | 1 | DAC, HSCT |
| 39 | m | 46 | AML with recurrent genetic abnormalities | 46,XY,inv(16)(p13.1q22) [20] | No | 0 | 11.95 | 38 | Intensive chemo, dasatinib |
| 41 | m | 53 | AML with recurrent genetic abnormalities | 46,XY [21] | No | 0 | 60.98 | 62 | Induction chemo, midostaurin, HSCT |
| 42 | f | 63 | AML-MRC | 44,XX,-3,-4,del(7)(q11),der(10) (:p13>10q26::?::17q21>17qter),del(12)(q15),der(13) t(3;13)(q13;q14),del(16)(q24),-17,-17,der(18) t(17;18)(q21;q21),+3mar [19]/46,XX [1] | No | 0 | 1.18 | 32 | Induction chemo, HSCT |
| 43 | f | 81 | AML, NOS | 46,XX [20] | No | 0 | 0.84 | 4 | Guadecitabine |
| 44 | m | 53 | AML-MRC | 46,XY [21] | No | 0 | 10.56 | 48 | Induction chemo, HSCT |
| 45 | m | 78 | AML-MRC | 46,XY [20] | No | 0 | 17.46 | 39 | HU |
| 46 | f | 57 | AML with recurrent genetic abnormalities | 46,X,-X,+8,del(9)(q21q34),t(15;17)(q24;q21) [20] | No | 0 | 40.76 | 49 | Intensive chemo, ATRA |
| 48 | f | 73 | AML with recurrent genetic abnormalities | 46,XX* | No | 0 | 1.64 | 2 | Intensive chemo |
| 52 | m | 53 | AML with recurrent genetic abnormalities | 46,XY,t(8;21)(q22;q22) [19]/46,XY [1] | No | 0 | 5.24 | 33 | Intensive chemo |
| 55 | m | 78 | AML, NOS | 46,XY [20] | No | 0 | 5.74 | 47 | DAC, ATRA, HU |
| 56 | m | 35 | AML with recurrent genetic abnormalities | 46,XY,t(9;16)(p10;p10)inv(16)(p13.1q22) [20], ish t(9;16)(p10;p10)(wcp9+,5`CBFB+;wcp9+,3`CBFB+) [6].nuc ish 16q22(CBFBx2)(5`CBFB sep 3`CBFBx1) [178/200] | No | 0 | 192.6 | 90 | Induction chemo, dasatinib, HSCT |
| 57 | f | 64 | AML with recurrent genetic abnormalities | 46,XX [21] | No | 0 | 11.7 | 58 | DAC, HSCT |
| 58 | m | 57 | AML with recurrent genetic abnormalities | 46,XY,del(11)(q21),t(15;17)(q24.1;q21.2) [12]/47,idem,+8 [8]/46,XY [1] | No | 0 | 0.94 | 52 | Arsenic trioxide, ATRA |

AML, acute myeloid leukemia; ATRA, all-trans retinoic acid; AZA, azacitidine; DAC, decitabine; FISH, fluorescence *in situ* hybridization; HSCT, hematopoietic stem cell transplantation; HU, hydroxyurea; LDAC, low dose cytarabine; MRC, myelodysplasia-related changes; N/A, not assessed; NOS, not otherwise specified; PB, peripheral blood; t-AML, therapy-related AML; VPA, valproic acid; WBC, white blood cells. *FISH analysis. †Deletion of *EZH2* gene confirmed by FISH.

**Supplementary Table 2: List of antibodies used for western blots**

| **Antibody** |  |
| --- | --- |
| Anti-EZH2, #612667 | *BD Biosciences* |
| Anti-H3, #9715 | *Cell Signaling Technology* |
| Anti-H3K27me3, #9733 | *Cell Signaling Technology* |
| Anti-b-Actin  Clone AC-74, #A2228 | *Sigma-Aldrich* |
| Anti-Rabbit IgG (H+L), Alexa Fluor Plus 800, #A32735 | *Invitrogen* |
| Anti-Mouse IgG (H+L), Alexa Fluor 680, #A21057 | *Invitrogen* |

**Supplementary Table 3: Primer sequences**

| **Gene** | | **Forward primer 5´-3´** | **Reverse primer 5´-3´** | **Sequencing primer 5´-3´** |
| --- | --- | --- | --- | --- |
| *EZH2* | | GTTTGATTGGGTTGGGGGGGTTAAAT | biotin-GTGCCAGGCTCAGGCCAAACTCCACTACCTTCTAA | GGGGGGTTAAATAAAAG |
| *p15/*  *CDKN2B*  *(1)* | Amplicon 1 | GTTGGTTTTTTATTTTGTTAGAG | biotin-AACTCAACTTCATTACCCTCC | GGGGTAGTGAGGATTT |
|  |  |  |  | TTTTTTAGAAGTAATTTAGG |
|  | Amplicon 2 | GAGGGTAATGAAGTTGAGTTTAGGTTT | biotin-CCAAAAACTATCRCACCTTCTCCA | TTTTAGGAAGGAGAGAGTG |
|  |  |  |  | GGTTAAYGGTGGATTATT |
|  |  |  |  | ATGAGGGTTTGGTTAG |
| *NY-ESO-1*  *(2)* | | TGGTTTAGGAGGTTTTGGTATTT | biotin-CATCTACAACATCCATTCAA | GGTTTAGGGGGTAATGT |

**Supplementary References**

1. Brakensiek K, Wingen LU, Langer F, Kreipe H, Lehmann U. Quantitative high-resolution CpG island mapping with Pyrosequencing reveals disease-specific methylation patterns of the CDKN2B gene in myelodysplastic syndrome and myeloid leukemia. Clin Chem. 2007;53(1):17-23.

2. Woloszynska-Read A, Mhawech-Fauceglia P, Yu J, Odunsi K, Karpf AR. Intertumor and intratumor NY-ESO-1 expression heterogeneity is associated with promoter-specific and global DNA methylation status in ovarian cancer. Clin Cancer Res. 2008;14(11):3283-90.

**Supplementary Figure Legends**

**Supplementary Figure 1:** Kaplan-Meier survival estimates in AML and MDS/MPN patients according to EZH2 expression and type of treatment.

(A) Low EZH2 protein expression is associated with a significantly shorter OS in the 41 intensively treated AML and MDS/MPN patients. 21 patients in the EZH2-high and 3 patients in the EZH2-low group were censored (still alive at last follow-up).

(B) In the 16 AML and MDS/MPN patients having received non-intensive therapy, OS is similar in patients with high or low EZH2 expression.

**Supplementary Figure 2:** Serial, on-treatment methylation analyses of the *EZH2* promoter of 6 decitabine (DAC)-treated MDS patients with (n=2) or without (n=4) monosomy 7.

DNA methylation levels of 11 CpGs located within the *EZH2* promoter were determined by bisulfite pyrosequencing prior to treatment (week 0), and at different time points during DAC treatment. Four-digit numbers represent DNA patient samples. Continuous color gradients represent methylation values between 0% (grey) and 100% (blue). Mean DNA methylation is indicated for each sample.
