# Supplementary figures and images for: Integrative study of EZH2 mutational status, copy number, protein expression and H3K27 trimethylation in AML/MDS patients
Source: Clin Epigenetics. 2021 Apr 12;13:77. doi: 10.1186/s13148-021-01052-2 (PMC8043064; doi:10.1186/s13148-021-01052-2)

## Slide 1
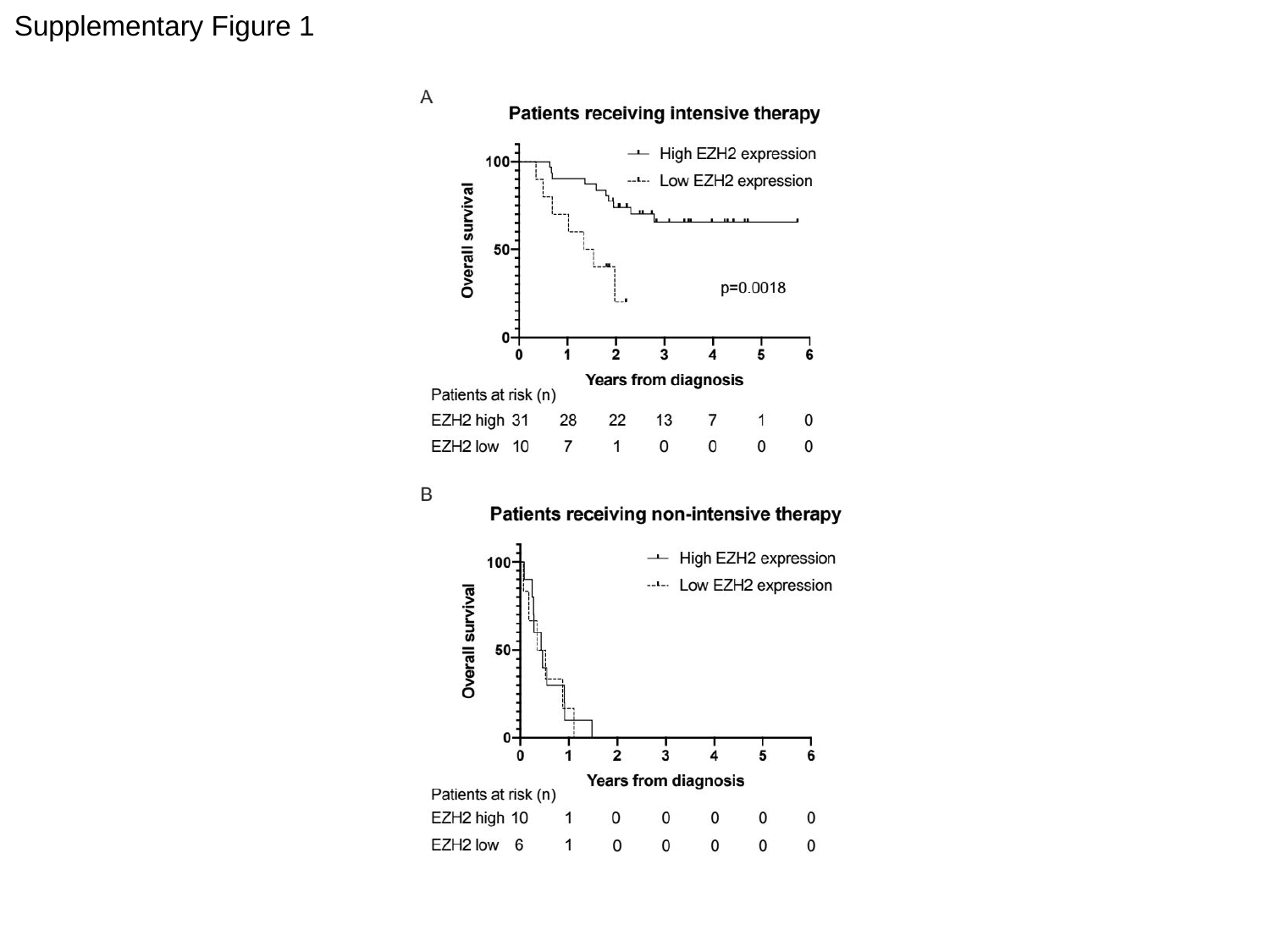

Supplementary Figure 1

## Slide 2
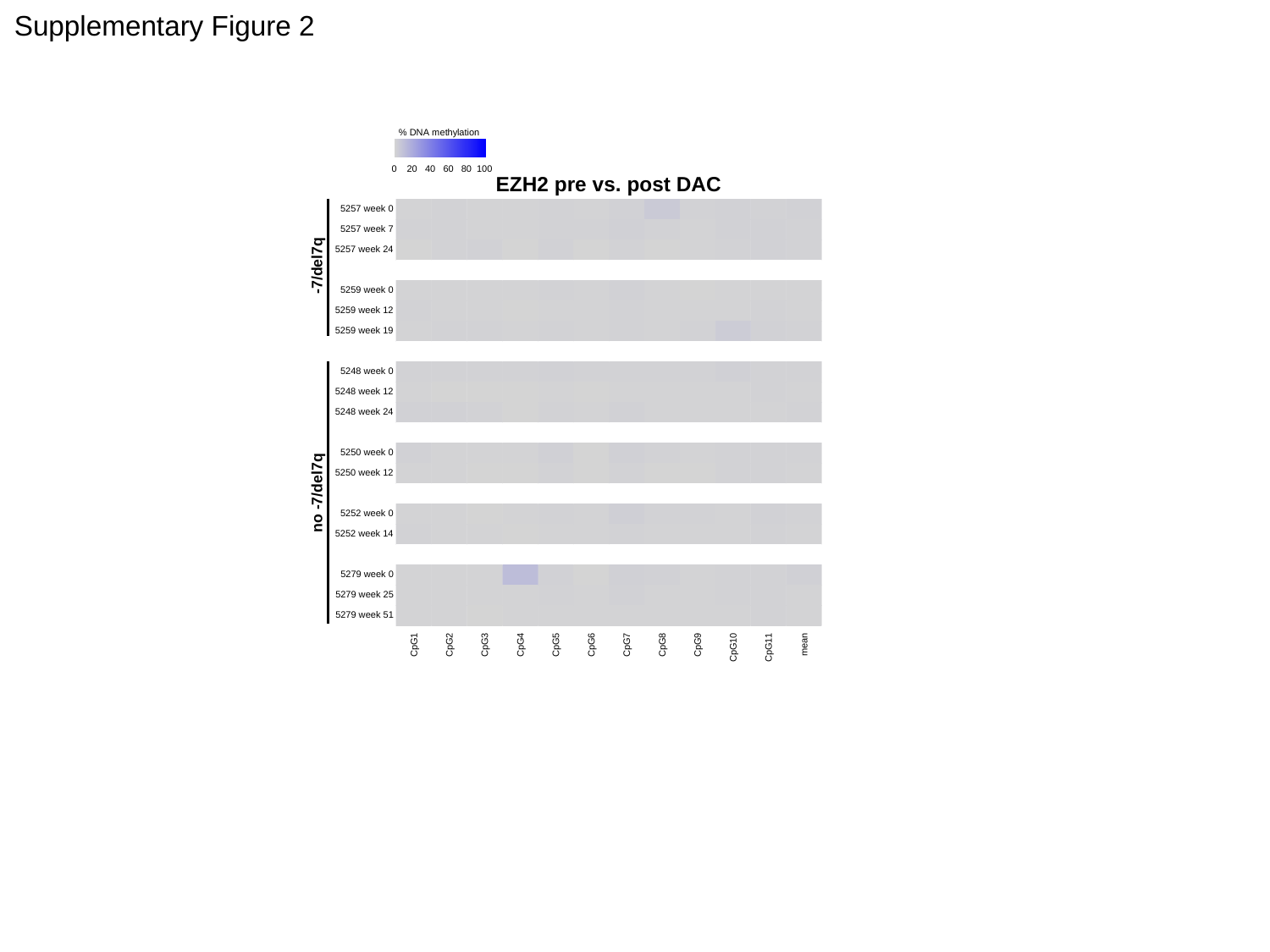

Supplementary Figure 2

Supplement: Supplementary file 2 — Additional file 2. Supplementary Figures. [file 13148_2021_1052_MOESM2_ESM.pptx]
